# Recent Research Advances in HER2-Positive Breast Cancer Concerning Targeted Therapy Drugs

**DOI:** 10.3390/molecules30143026

**Published:** 2025-07-18

**Authors:** Junmin Li, Xue Li, Ruixin Fu, Yakun Fang, Chunmei Zhang, Bingbing Ma, Yanan Ding, Chuanxin Shi, Qingfeng Zhou

**Affiliations:** 1 School of Biology and Food Science, Shangqiu Normal University, Shangqiu 476000, China; lijunmin5760@163.com (J.L.); ruixinfu2022@163.com (R.F.); fangyakun468@126.com (Y.F.); zcm304@163.com (C.Z.); mabingbing3062@163.com (B.M.); dingyanan13@163.com (Y.D.); 2 Basic Medical Department, Nanyang Medical College, Nanyang 473001, China; szw2003@126.com

**Keywords:** breast cancer, human epidermal growth factor receptor 2 (HER2), targeted therapy drugs, monoclonal antibodies (mAbs), tyrosine kinase inhibitors (TKIs), antibody-drug conjugates (ADCs), bispecific antibodies (bsAbs)

## Abstract

Breast cancer is one of the most common malignant tumors among women, which seriously threatens women’s health. Human epidermal growth factor receptor 2 (HER2)-positive breast cancer, characterized by poor prognosis, is an aggressive phenotype accounting for 15–20% of all kinds of breast cancers. Therefore, it has attracted great interest among researchers in discovering targeted therapy drugs countering HER2, and they have been considered as the pivotal therapeutic regimen for HER2-positive breast cancer patients. Nowadays, large progress has been achieved in HER2-targeted drugs, and this review categorizes them into four types according to the drug action mode, including monoclonal antibodies (mAbs), tyrosine kinase inhibitors (TKIs), antibody-drug conjugates (ADCs), and bispecific antibodies (bsAbs). The progress of HER2-targeted drugs reflects the discovery of drug targets, the screening of drug compounds, and the modification of antibodies, which offer diverse medical options and better therapeutic benefits for individual patients. In detail, we focus on the indication, administration, efficacy, strengths, and challenges of HER2-targeted drugs, concerning approved drugs and clinical trials. This review aims to provide significant references for the targeted therapeutic regimen and a more precise treatment strategy for HER2-positive breast cancer.

## 1. Introduction

Breast cancer has become one of the most common cancers among women and a major cause of cancer-related deaths, exerting a tremendous impact on women’s health worldwide. The risk factors, such as genetics, hormone exposure, unhealthy lifestyles, and medical interventions, may elevate the susceptibility of breast cancer [1]. In 2022, there were about 2.3 million new cases and 670,000 deaths of breast cancer among women worldwide; it is projected that by 2050, the number of new cases and deaths will increase by 38% and 68%, respectively, particularly in countries with a low human development index [2,3]. In view of the Global Burden of Disease database, China reported over 3 million cases of breast cancer in 2021, with the incidence rate doubling compared to 1990 [4]. Furthermore, multicenter studies have shown that the proportion of young breast cancer patients in China significantly exceeds Western countries [5]. Based on the gene expression and protein markers of estrogen receptor (ER), progesterone receptor (PR), and human epidermal growth factor receptor 2 (HER2), the molecular phenotypes of highly heterogeneous breast cancer can be categorized into Luminal A (ER^+^/PR^+^, HER2^−^, low Ki-67), Luminal B (ER^+^/PR^+^, HER2^−^, high Ki-67; ER^+^/PR^+^, HER2^+^), HER2-positive (ER^−^/PR^−^, HER2^+^), and triple negative breast cancer (TNBC, ER^−^/PR^−^/HER2^−^), different molecular phenotypes have different epidemiology and diagnostic criteria, and they are pivotal for the individual treatment options and prognosis evaluation of breast cancer [6].

HER2, also known as ERBB2, is one of the members of the epidermal growth factor receptor (EGFR) family and belongs to the transmembrane glycoprotein-tyrosine kinase receptor (RTK), which forms heterodimers with other EGFR family members (HER1, HER3, HER4) to activate downstream signaling pathways (such as PI3K/AKT/mTOR, RAS/MEK/ERK, JAK/STAT, etc.) and regulate cellular proliferation, differentiation, survival [7]. The overexpression or gene amplification of HER2 can lead to the hyperactivation of the receptor-mediated signal transduction and promote the tumorigenesis of breast cancer. HER2-positive occurs in 15–20% of the four molecular phenotypes above, which has attracted much attention due to its strong invasiveness and poor prognosis [8]. In China, the proportion of HER2-positive breast cancer is basically consistent with the global data. The oncology team from Fudan University further classified HER2-positive breast cancer into four subtypes through multi-omics analysis, involving a classical HER2 subtype, immunomodulatory subtype, luminal-like subtype, and basal/mesenchymal-like subtype [9].

For the distinct molecular phenotypes of breast cancer, medical options usually include surgical operation and radiotherapy, endocrine therapy, systemic therapy (chemotherapy, targeted therapy, immunotherapy), and emerging therapies. In response to HER2-positive breast cancer, the drugs targeting HER2 have been greatly developed and are available for therapeutic regimen nowadays, including monoclonal antibodies (mAbs), tyrosine kinase inhibitors (TKIs), antibody-drug conjugates (ADCs), bispecific antibodies (bsAbs), and other novel drug administrations. Despite multiple approved therapies, resistance and recurrence remain major clinical challenges for HER2-positive breast cancer, due to HER2 alteration, tumoral heterogeneity, and alternative pathways. Thus, in this review, we introduce, in detail, the drug action mode, indication, administration, efficacy, strengths, and challenges of various HER2-positive breast cancer targeted therapy drugs, involving drugs approved and in clinical trials, in order to summarize the current targeted therapy drugs and therapeutic regimen for HER2-positive breast cancer, and provide references for more precise treatment strategy.

## 2. HER2 Signaling Pathway to Exacerbate Breast Cancer

**Figure 1:**
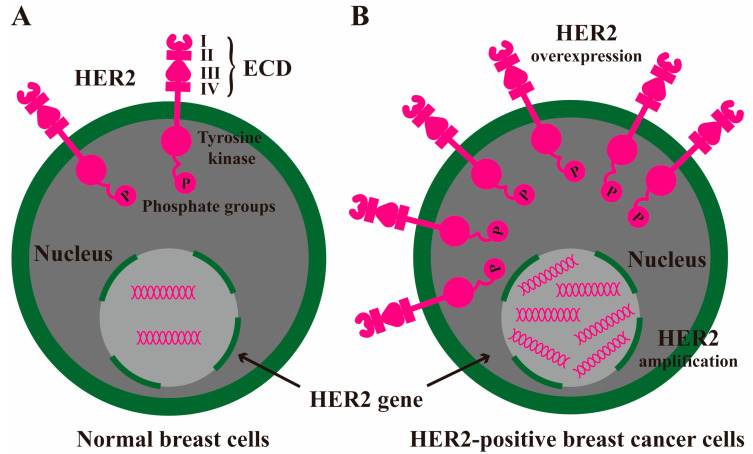
Gene expression pattern of HER2 in normal and tumor cells: (**A**) Normal breast cells. HER2 is lowly expressed or not expressed in breast epithelial cells. (**B**) HER2-positive breast cancer cells. HER2 is amplified or overexpressed in this phenotype of breast cancer. ECD I-IV: extracellular domain I–IV.

In 1979, Weinberg’s team extracted DNA from rat neuroblastoma and induced carcinogenesis into normal mouse cells. They found a special transmembrane protein encoded by rat genes and named it neu gene; later, scientists discovered that the structure of neu was similar to ERBB2/HER2 gene and successfully cloned HER2 and measured the complete sequence; in 1987, it had been found that there existed excessive HER2 amplification or overexpression in some breast cancer patients, leading to uncontrolled tumorigenesis, lower survival rates, and faster recurrence [10,11,12,13]. HER2 is lowly expressed or not expressed in the epithelial cells of normal skin, breast, respiratory tract, gastrointestinal tract, and urethra, while it is amplified or overexpressed in multiple tumors like breast cancer (Figure 1). As one of the genes that have been investigated more thoroughly in breast cancer to date, HER2 overexpression is not only linked to the occurrence and development of tumors, but also an ideal clinical treatment monitoring and prognostic indicator, and a specific drug candidate for tumor-targeted therapy. Therefore, studies on HER2 have long been valued by both the scientific and medical communities.

**Figure 2:**
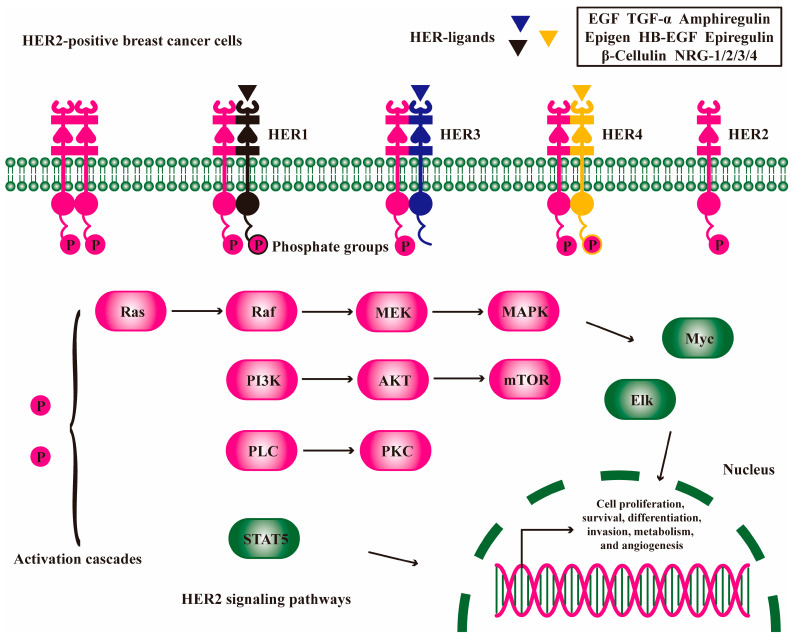
HER2 signaling pathway exacerbates breast cancer. The ECD ligands of the HER family promote HER2 homologous dimerization and heterologous dimerization with another HER1/3/4 to activate downstream Ras/Raf/MEK/MAPK, PI3K/AKT, PLC/PKC, and STAT5 signaling pathways, which mediate the transcription of genes related to cell proliferation, survival, differentiation, invasion, metabolism, and angiogenesis.

HER2 is an oncogene located on chromosome 17, and the tyrosine kinase receptor it encodes belongs to the epidermal growth factor receptor (EGFR) family containing HER1-4 [14]. These surface receptors are composed of an extracellular ligand-binding domain, a transmembrane domain, and an intracellular tyrosine kinase domain. The extracellular domain (ECD) consists of four subdomains, which are divided into two extracellular ligand-binding domains and two domains responsible for the receptor dimerization process [15]. The ligands of the HER family include EGF, transforming growth factor-α (TGF-α), amphiregulin, epigen, heparin-binding epidermal growth factor-like growth factor (HB-EGF), epiregulin, β-cellulin, neuregulin-1/2/3/4 (NRG-1/2/3/4), etc. [16,17,18,19]. After these ligands bind to HER, they cause changes in receptor conformation and promote a free receptor combination to form homodimers or heterodimers with different degrees of binding activity. However, HER2 has no extracellular ligand-binding domain and cannot combine with ligands. Its activation depends on homologous dimerization with another HER2 or heterologous dimerization with another HER1/3/4 [20]. Compared with homodimers, HER2 heterodimers have a higher ligand-binding ability and a stronger signal transduction ability. The autophosphorylation of receptor tyrosine residues in the cytoplasmic region acts as the docking site of other tyrosine kinases to initiate the signal cascade and mediate various biological effects [15,21]. Therefore, HER2 heterodimers form in large quantities and are the main reason for drug resistance and poor prognosis of HER2-positive breast cancer. The signaling pathways they activate are mainly Ras/Raf/MEK/MAPK, PI3K/AKT, PLC/PKC, and STAT5, which eventually activate downstream nuclear delivery genes such as MYC, ELK, JUN, and STAT5 to mediate the transcription of genes related to cell proliferation, survival, differentiation, invasion, metabolism, and angiogenesis (Figure 2) [22,23,24,25]. In addition, HER2 dimerization promotes the transcription and degradation of p27Kip1 protein, a cell cycle inhibitor, thereby accelerating the cell cycle process and tumor cell proliferation [26]. Meanwhile, HER2 can also interact with other tyrosine kinase receptors, extracellular matrix receptors, and hepatocyte growth factor receptor to activate downstream signal transduction via alternative pathways [27,28,29].

Thus, it can be seen that the excessive cell proliferation caused by HER2 overexpression eventually promotes the growth and metastasis of tumors. This mechanism has been fully investigated and demonstrated in breast cancer. HER2 gene amplification is one of the most critical factors exacerbating the growth and metastasis of breast cancer and resulting in poor prognosis. Breast cancer patients with overexpressed HER2 have rapid disease progression, a short chemotherapy remission period, poor effect of endocrine therapy, low disease-free survival, and overall survival (OS) rate. Therefore, the content of HER2 can serve as an independent and powerful indicator for the diagnosis, treatment, and prognosis of breast cancer in clinical practice. The development of monoclonal antibodies targeting the ligand-binding domain has changed the medical landscape of HER2-positive breast cancer. Given that all three domains of HER2 play significant roles in signal transduction, each domain can be independently targeted to block HER2 signaling. With the progress of HER2-targeted drugs, the therapeutic benefits have expanded from HER2-positive patients with low HER2 expression. However, some patients may develop drug resistance, and HER2 alteration (low expression, high heterogeneity, mutation, modification, truncation, dimerization), tumoral heterogeneity, downstream signal mutation, and alternative pathways may be one of the potential mechanisms of acquired resistance in HER2-targeted therapy. Therefore, the treatment of HER2-positive breast cancer patients with drug resistance needs to focus on previous medication history, tumor burden, and molecular characteristics. In the future, with the advanced application of new drugs, the improvement of individualized treatment strategies and targeted combination regimens is expected to further prolong the survival period of patients.

## 3. mAb Drugs

**Figure 3:**
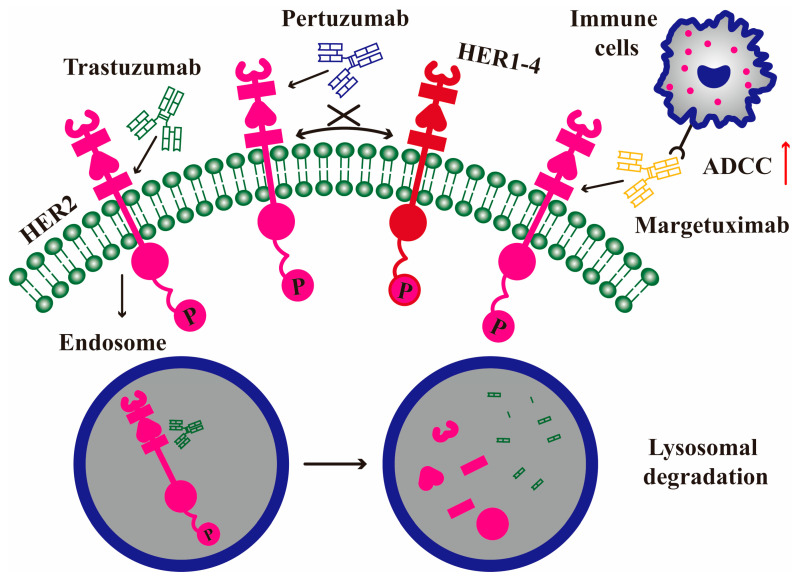
The action mode of mAb drugs blocks HER2 signaling. MAbs bind to the ECD II/IV of HER2 to block the HER2 dimerization and trigger HER2 endocytosis. The endocytosed HER2 is degraded from the endosome to the lysosome. The Fc fragment of mabs can recruit extracellular immune cells to perform the ADCC effect. The red arrow represents enhancing effect.

After decades of research and development, mAb drugs have demonstrated unique advantages and broad application prospects in the field of oncology due to their high specificity and definite therapeutic effects. At present, mAb drugs have become one of the most effective strategies of tumor therapy, and HER2 is a popular target for mAb drugs treating breast cancer (Table 1). The action mode of mAb drugs involves two aspects (Figure 3): binding to the ECD of HER2 to prevent the formation of HER2-containing heterodimers and regulate downstream signal transduction; recruiting extracellular immune cells to perform antibody-dependent cell-mediated cytotoxicity (ADCC) [30].

### 3.1. Trastuzumab

Trastuzumab (Herceptin), as the global first humanized mab targeting HER2, was developed by Genentech and approved by the Food and Drug Administration (FDA, Silver Spring, MD, USA) in 1998 for the treatment of HER2-positive metastatic breast cancer patients who had previously received chemotherapy. Trastuzumab inhibits the homologous or heterologous dimerization of HER2 by specifically binding to the ECD IV, thereby blocking the activation of downstream signaling pathways and inhibiting the survival and angiogenesis of tumor cells [31]. After trastuzumab binds to HER2, it induces the receptor-antibody complex to enter the lysosomal degradation pathway through endocytosis, reducing the expression of HER2 on the cell surface and thereby weakening its cancer-promoting activity [32]. The Fc segment of trastuzumab binds to the Fcγ receptor on the surface of immune cells, recruiting them to attack and kill HER2-positive breast cancer cells [33]. The clinical trial of Slamon DJ et al. evaluated the efficacy and safety of trastuzumab and showed that trastuzumab could significantly improve the symptoms and prolong the OS of HER2-positive advanced breast cancer patients, making trastuzumab + chemotherapy the standard regimen for HER2-positive advanced breast cancer [34]. Later, the FDA approved trastuzumab for operable adjuvant treatment of early breast cancer to improve the rate of invasive-disease-free survival and reduce the risk of recurrence [35]. In recent years, to reduce treatment costs and improve drug accessibility, the biosimilars of trastuzumab have been approved to treat HER2-positive breast cancer, such as anqutuo, herzuma, trazimera, tuznue, and so on [36,37,38,39]. Through comprehensive preclinical studies, these biosimilars have been confirmed to be highly comparable to the original Herceptin in terms of physicochemical properties, HER2-binding activity, HER2 signal blocking, anti-proliferative ability, immunomodulatory function, and safety.

Drug resistance is the main restriction factor for the clinical usage of trastuzumab in HER2-positive breast cancer patients. The exfoliated fragments of HER2 ECD contain trastuzumab recognition epitopes and carcinogenic fragments with sustained kinase activity, which can promote tumor survival [40]. Activation mutations, expression decline, or the modification of HER2 can change receptor conformation and downstream signal transduction, reducing the binding efficiency of trastuzumab [41]. In addition, the loss of PTEN, mutations of Ras/Raf/MEK/MAPK, and PI3K/AKT can overactivate downstream signaling pathways; alternative signals such as insulin-like growth factor 1 receptor (IGF-1R), other heterodimerized EGFR family members will compensatorily activate downstream signaling pathways [42,43,44]. Thus, the combined action of multiple mechanisms leads to trastuzumab resistance, inhibiting overactivated signaling pathways, multiple targeting, and feasible combination strategies offer breakthroughs to solve the resistance of mAb drugs.

### 3.2. Pertuzumab

Based on trastuzumab targeting, the ECD IV of HER2, scientists have discovered that HER2 needs to activate downstream signaling pathways by forming heterodimers with other ERBB family members, such as HER3. Pertuzumab (Perjeta), developed by Genentech, targets the ECD II of HER2 and blocks its dimerization with other receptors, thereby more comprehensively inhibiting HER2 signal complement with trastuzumab [45]. In 2012, the NeoSphere trial demonstrated that the pathological complete response rate of the neoadjuvant therapy of pertuzumab combined with trastuzumab and docetaxel achieved a significantly higher rate than the single trastuzumab group; the CLEOPATRA trial demonstrated that pertuzumab + trastuzumab + chemotherapy in the treatment of patients with metastatic HER2-positive breast cancer extended the median OS and reduced the risk of death compared with the control group [46,47]. These clinical trial results accelerated the FDA approval of pertuzumab and established the standard of pertuzumab + trastuzumab + chemotherapy in the first-line treatment of patients with HER2-positive metastatic breast cancer and the adjuvant treatment of HER2-positive early breast cancer. Pertuzumab has different binding sites and action mode from trastuzumab and can work synergistically with trastuzumab to exert anti-tumor effects. Moreover, in 2021, the FeDeriCa trial demonstrated that pertuzumab + trastuzumab + hyaluronidase in the subcutaneous injection of patients with HER2-positive early breast cancer could significantly shorten the administration time with similar pathological complete response rate and pharmacokinetic characteristics to intravenous preparation [48]. TQB-2440, developed by Chia Tai Tianqing Pharmaceutical Group, was approved in China in 2024, becoming the first globally approved pertuzumab biosimilar, and clinical trials demonstrated that it is equivalent to the original drug in terms of pathological complete response rate and safety [49]. Other biosimilars applied for approval or in clinical trials include QL1209 (NCT04629846) and HLX11 (NCT04411550), which exhibited equivalent efficacy and comparable safety to the original pertuzumab [50,51].

### 3.3. Margetuximab

The first HER2-targeted drug, trastuzumab, can significantly improve the prognosis of patients with HER2-positive breast cancer; however, some patients will relapse due to drug resistance or insufficient immune response, for instance, the low activity of NK cell-mediated ADCC. Thus, insufficient affinity between the Fc segment and the activated receptor of ADCC is the crucial factor limiting mAb drugs. MacroGenics has modified the Fc segment of margetuximab (Margenza) through antibody engineering to enhance its affinity to the activated Fcγ receptor CD16A and reduce its affinity to the inhibitory receptor CD32B, thereby improving the NK cell killing activity and ADCC effect [52]. The SOPHIA trial demonstrated that in patients with metastatic HER2-positive breast cancer who had previously received anti-HER2 therapy, the chimeric antibody margetuximab + chemotherapy, over trastuzumab + chemotherapy, improved progression-free survival (PFS) with a median of 5.7 months vs. 4.4 months, median OS with 21.6 months vs. 19.8 months, and objective response rate with 25% vs. 14%, accompanied by comparable safety [53]. In 2020, the FDA approved margetuximab + chemotherapy for the subsequent therapy of metastatic HER2-positive breast cancer. The patients carrying the CD16A allele were more likely to benefit from the margetuximab drug; therefore, genetic screening before medication is very important for the precise treatment.

**Table 1:** The mAb drugs for HER2-positive breast cancer.

MAb Drugs	Phase	Action Mode	Refs.
Trastuzumab (Herceptin)	Approved	Binding to ECD IV, blocking HER2 dimerization, HER2 endocytosis; ADCC	[31,32,33,34,35]
Anqutuo Herzuma Trazimera Tuznue	Approved	Trastuzumab biosimilars	[36,37,38,39]
Pertuzumab (Perjeta)	Approved	Binding to ECD II, blocking HER2 dimerization; ADCC	[45,46,47]
TQB-2440 QL1209 HLX11	Approved Applied for approval (NCT04629846) Phase I (NCT04411550)	Pertuzumab biosimilars	[49,50,51]
Margetuximab (Margenza)	Approved	Binding to ECD IV, blocking HER2 dimerization; enhancing Fc-CD16A and reducing Fc-CD32B to improve ADCC	[52,53]

## 4. TKI Drugs

**Figure 4:**
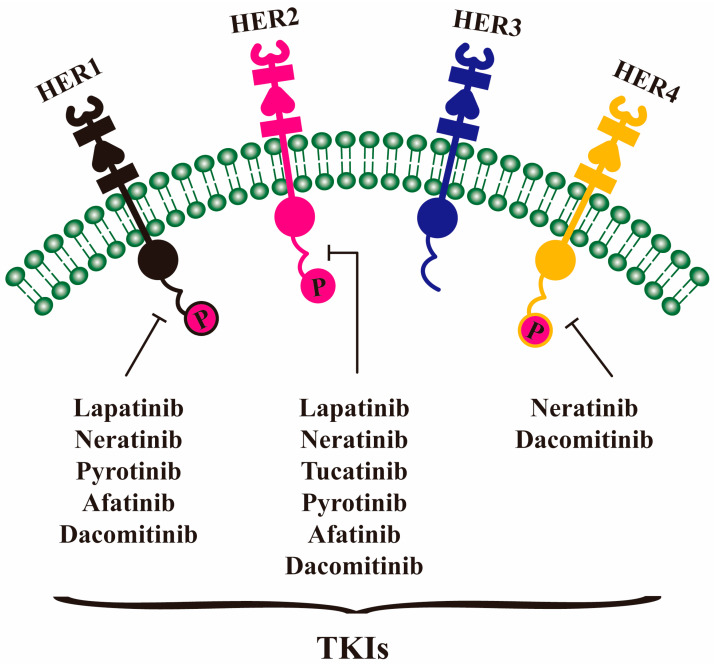
The action mode of TKI drugs blocks HER2 signaling. TKIs bind to the kinase ATP binding site of the intracellular tyrosine kinase domain of HER1/2/4 to suppress phosphorylation and downstream signal transduction. Targeting HER1: lapatinib, neratinib, pyrotinib, afatinib, dacomitinib; targeting HER2: lapatinib, neratinib, tucatinib, pyrotinib, afatinib, dacomitinib; targeting HER4: neratinib, dacomitinib.

TKI drugs are a class of small-molecule drugs that target tyrosine kinase activity to block abnormal signal transduction. They are widely employed in cancer treatment to restrain tumor growth and trigger apoptosis by competitively or irreversibly binding to the kinase ATP binding site, suppressing phosphorylation and downstream signal transduction [54]. The approved HER2 small molecule inhibitors have significantly improved the prognosis of breast cancer patients with drug resistance and distant metastasis by targeting the key nodes of the related signaling pathway, which are categorized in Table 2 together with the drugs in clinical trials (Figure 4).

### 4.1. Lapatinib

To overcome the drug resistance problem of trastuzumab, GSK developed an oral and dual TKI lapatinib (Tykerb), a 4-aniline quinazoline small molecule compound (C_29_H_26_ClFN_4_O_4_S, 581.06), which simultaneously targets HER1 and HER2 to block their downstream signaling pathways and inhibit tumor growth by blocking the binding of ATP to kinases. Otherwise, the inactivated AKT and MAPK signaling pathways may trigger dephosphorylation to reduce cyclin-dependent kinase 4/6 (CDK 4/6)-cyclin D activity, activate pro-apoptotic proteins, and inactivate anti-apoptotic proteins, leading to tumor cell cycle arrest and apoptosis [55,56]. For the patients with HER2-positive, locally advanced or metastatic breast cancer that had progressed after the treatment regimen of thracycline, taxane, and trastuzumab, the trial of Geyer CE et al. demonstrated that the median progression time was 8.4 months in the lapatinib + capecitabine group as compared with 4.4 months in the monotherapy group [57]. In 2007, the FDA approved lapatinib + capecitabine for HER2-positive metastatic breast cancer that failed trastuzumab treatment, which became the first approved HER2-targeted TKI. Subsequently, the treatment regimen of lapatinib + paclitaxel also achieved similar high percentages of pathological complete response in substitution of trastuzumab [58]. The discovery of lapatinib marks the entry of HER2-positive breast cancer treatment into the TKI era. Although abnormal HER2, alternative signal activation, and metabolic reprogramming will occur in the long-term administration of lapatinib, it is still included in studies of combination regimens due to oral convenience and the activity of penetrating the blood–brain barrier against brain metastasis [59,60].

### 4.2. Neratinib

Puma Biotechnology developed an oral neratinib (C_30_H_29_ClN_6_O_3_, 557.04), composed of pyridine methoxy aniline, butenamide, cyano group, and ethoxy group, to prevent the excessive transduction of HER1, HER2, and HER4 signaling pathways through irreversible binding, aiming to overcome the drug resistance of mAb drugs such as trastuzumab [61,62]. The ExteNET trial demonstrated that neratinib-extended adjuvant therapy reduced relapse and improved invasive disease-free survival (iDFS, 90.2% vs. 87.7%) in women with HER2-positive breast cancer and after trastuzumab adjuvant therapy; thus, the FDA approved neratinib for extended adjuvant therapy in early HER2-positive breast cancer [63]. In metastatic HER2-positive breast cancer, the NALA trial compared neratinib+capecitabine and lapatinib +capecitabine, which verified that patients who previously received at least two HER2-targeted treatments had extended PFS in the neratinib group, and no new safety signals were observed [64]. Then, the FDA approved neratinib for the third-line treatment of HER2-positive metastatic breast cancer. Although diarrhea is associated with the early discontinuation of neratinib, diarrhea incidence can be reduced after the precaution of loperamide and colestipol [65,66].

### 4.3. Tucatinib

Tucatinib (C_26_H_24_N_8_O_2_, 480.52), developed by Seattle Genetics, is an oral, reversible ATPase-competitive HER2 inhibitor, in which the core structure is a quinazoline derivative. In cell lines with HER2 overexpression, tucatinib blocks the phosphorylation of AKT and cell proliferation; conversely, in cell lines with EGFR overexpression, it weakly inhibits phosphorylation and proliferation, indicating that tucatinib may have the potential to selectively block HER2 signaling without causing EGFR inhibitory toxicity [67]. Patients with HER2-positive metastatic breast cancer are prone to drug resistance after multiple lines of treatment and urgently need new targeted drugs. The highly selective HER2 TKI tucatinib can penetrate the blood–brain barrier and improve brain metastasis. In the HER2CLIMB trial, patients with HER2-positive metastatic breast cancer previously treated with trastuzumab, pertuzumab, and trastuzumab emtansine received tucatinib + trastuzumab + capecitabine, and the results demonstrated that the median PFS extended 2.2 months and the median OS extended 4.3 months [68]. In 2020, the FDA approved tucatinib + trastuzumab + capecitabine for ≥second-line treatment of advanced or metastatic HER2-positive breast cancer, covering brain metastasis. Unlike other pan-HER inhibitors (lapatinib, neratinib), tucatinib has an extremely low inhibitory effect on EGFR, which will reduce off-target toxicities such as rash and diarrhea [69].

### 4.4. Pyrotinib

Pyrotinib (C_32_H_31_ClN_6_O_3_, 583.08), developed by Hengrui Medicine, was approved by the Chinese FDA in 2018, with the indication of HER2-positive advanced breast cancer. It is a novel oral and irreversible HER1/HER2 dual TKI with a favorable safety profiles for the treatment of HER2-positive breast cancer [70]. The PHOEBE trial compared the efficacy of pyrotinib + capecitabine and lapatinib + capecitabine in patients with HER2-positive metastatic breast cancer, who previously received trastuzumab therapy, and indicated a better PFS to pyrotinib (5.9 months extended) [71]. The PHILA trial demonstrated a benefit in terms of PFS derived from the addition of pyrotinib to first-line trastuzumab + chemotherapy in patients with metastatic HER2-positive breast cancer; however, the increasing risk of gastrointestinal toxicity should be carefully assessed [72]. The following research verified that pyrotinib + chemotherapy remained effective for breast cancer patients with low HER2 expression [73]. The research and development process of pyrotinib reflects the gradual expansion from second-line treatment for advanced breast cancer to first-line and neoadjuvant therapy. The continuous broadening of indications has further consolidated its role in the treatment of HER2-positive breast cancer. More clinical trials are still ongoing and may lead to the approval of more indications in the future.

### 4.5. Afatinib and Dacomitinib

Afatinib and dacomitinib are irreversible EGFR TKIs for HER1/2 and HER1/2/4, and are designed to overcome the drug resistance of the first-generation reversible EGFR TKIs and persistently inhibit the receptor signaling pathway through irreversible binding. Although early studies of afatinib and dacomitinib focused on other cancers like lung, their inhibitory effect on HER2 has triggered preclinical and clinical exploration for HER2-positive breast cancer. A phase 3 trial did not find that the efficacy of afatinib was superior to trastuzumab, and a cell experiment showed that dacomitinib maintained a high activity in HER2-amplified breast cancer lines resistant to trastuzumab and lapatinib [74,75]. Therefore, the research and development of afatinib and dacomitinib in HER2-positive breast cancer is still in the exploration stage, and their potential may lie in combined therapy or precise application in patients with specific molecular phenotypes.

**Table 2:** The TKI drugs for HER2-positive breast cancer.

TKI Drugs	Phase	Targets	Refs.
Lapatinib	Approved	HER1, HER2	[55,56,57,58]
Neratinib	Approved	HER1, HER2, HER4	[61,62,63,64,65]
Tucatinib	Approved	HER2	[67,68]
Pyrotinib	Approved	HER1, HER2	[70,71,72,73]
Afatinib	Phase III (NCT01125566)	HER1, HER2	[74]
Dacomitinib	Preclinical	HER1, HER2, HER4	[75]

## 5. ADC Drugs

**Figure 5:**
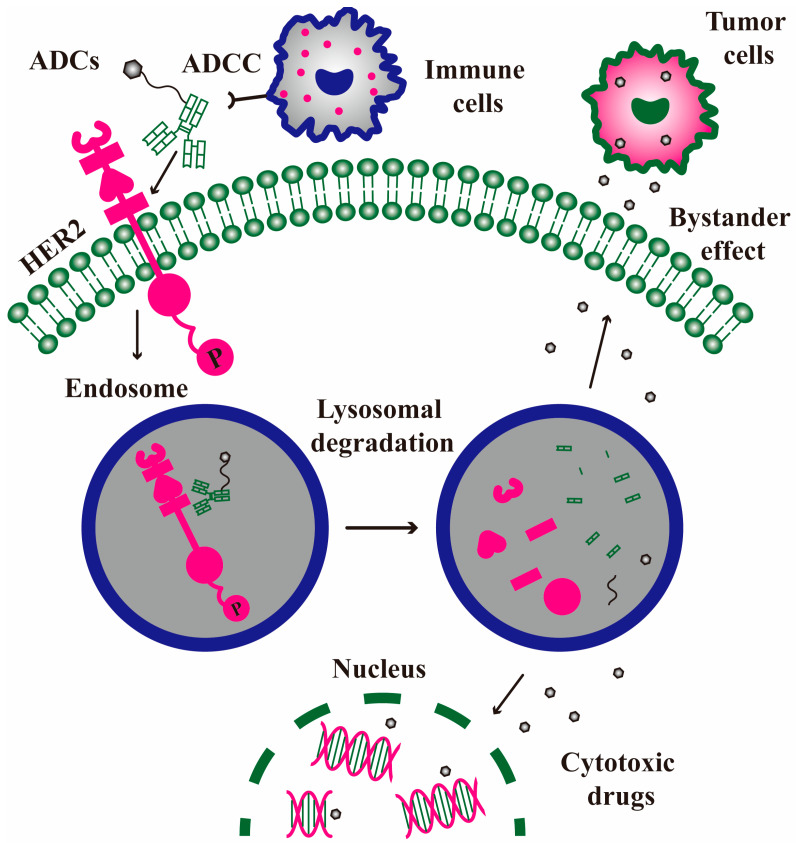
The action mode of ADC drugs blockading HER2 signaling. ADCs bind to HER2 and trigger HER2 endocytosis. The endocytosed HER2 plus ADCs are degraded from the endosome to the lysosome, thereby releasing cytotoxic drugs to kill tumor cells (bystander effect or not). Moreover, the Fc fragment of ADCs can recruit extracellular immune cells to perform the ADCC effect.

ADC drugs are composed of mAbs, small-molecule cytotoxic drugs, and linkers, containing the powerful killing effect of traditional small-molecule chemotherapy and tumor-targeting property. The recognition of antigens leads to the endocytosis of ADC, and the loading drugs are released in a bioactive form after lysosome degradation, resulting in tumor cell death effects [76]. The number of intracellular payloads is determined by the number of surface antigens per cell, the number of drug payload molecules per ADC (drug-antibody ratio, DAR), and the time required for the antigens to return to the cell surface (Figure 5). The ADC drugs approved and in clinical trials are categorized in Table 3.

### 5.1. Trastuzumab Emtansine

In 2013, the FDA approved trastuzumab emtansine (T-DM1) developed by Roche Group for HER2-positive breast cancer. This drug couples the tubulin inhibitor DM1 to the lysine residue of trastuzumab through an uncleavable thioether linker. Compared with the first-generation ADC drugs, T-DM1 uses a fully humanized IgG1 antibody with low immunogenicity; the payload DM1 with higher toxicity improves the water solubility and coupling efficiency without antibody gathering, achieves better plasma stability, and a more uniform distribution of DAR. But the internalized metabolite of T-DM1 cannot pass through the cell membrane at physiological pH, resulting in no bystander effect [77]. The EMILIA trial assigned patients with HER2-positive advanced breast cancer, who had previously been treated with trastuzumab + taxane, to T-DM1 or lapatinib + capecitabine, and demonstrated that T-DM1 extended median PFS and OS for 3.2 and 6.8 months, with fewer side effects than lapatinib [78]. The KATHERINE trial assigned patients with HER2-positive early breast cancer who had residual invasive disease in the breast or axilla at surgery after receiving adjuvant therapy containing taxane + trastuzumab to receive adjuvant T-DM1 or trastuzumab, and demonstrated that T-DM1 reduced by 50% to the risk of recurrence of invasive breast cancer or death but displayed more associated adverse events [79]. The long-term follow-up data showed that the 7-year iDFS and OS in the T-DM1 group significantly increased by 13.7% and 4.7% compared with the control group [80].

### 5.2. Trastuzumab Deruxtecan

The representative drug of the third-generation ADC is trastuzumab deruxtecan (T-DXd), which was developed by AstraZeneca/Daiichi Sankyo and approved for breast cancer by the FDA in 2019. It is composed of a humanized anti-HER2 mAb, a DNA topoisomerase inhibitor, DXd, and a stable, cleavable tetrapeptide linker. Compared with T-DM1, T-DXd adopts a stable hydrophilic linker in systemic circulation, which can precisely control drug release to the tumor site; the toxin of the new mechanism improves drug resistance, and T-DXd has good cell membrane permeability, which can not only kill HER2-positive tumor cells, but also kill adjacent tumor cells through the bystander effect [81]. The clinical trials had demonstrated the superior efficacy and low resistance of T-DXd over T-DM1 in the treatment of patients with advanced HER2-positive metastatic breast cancer, with median PFS 29.0 vs. 7.2 months, median OS 52.6 vs. 42.7 months, and a manageable safety profile [82,83,84]. T-DXd has replaced T-DM1 as the new standard for the second-line treatment of HER2-positive breast cancer with high efficacy and long-lasting remission. Clinical trials for HER2-low and HER2-negative breast cancer are underway, aiming to expand the beneficiary population [85].

### 5.3. Other ADC Drugs and Bystander Effect

After three generations of technological upgrades, ADC drugs have continuously made breakthroughs in research progress, such as target, antibody, linker, payload, and coupling method. In recent years, the application of ADC drugs in breast cancer therapy has become increasingly widespread, especially HER2 ADC drugs have displayed significant efficacy in the second-line and late-line treatments of advanced breast cancer, becoming a research hotspot in this field. Other ADC drugs, such as RC48: hertuzumab + valine-citrulline linker + tubulin inhibitor monomethyl auristatin E (MMAE), A166: trastuzumab + valine-citrulline linker + tubulin inhibitor duostatin-5 (Duo-5) and SHR-A1811: trastuzumab + tetrapeptide linker + topoisomerase inhibitor SHR9265 mainly remain at clinical trials, and demonstrate considerable therapeutic effects in the treatment of HER2-positive breast cancer [86,87,88].

The payload may escape after the death and degradation of cancer cells, or it may penetrate through the membrane from the cytoplasm (Figure 5). The bystander effect can spread the payload from antigen-positive tumor cells to adjacent antigen-negative tumor cells, thereby enhancing anti-tumor activity. This type of ADC drug requires cleavable linkers and hydrophobic payloads that can penetrate through the cell membrane to achieve drug diffusion. But the consequences of the bystander effect may be beneficial, or they may be harmful, since the bystander effect may spread the payload to adjacent normal tissues, thereby causing systemic toxicity. The bystander effect depends on the heterogeneous expression of target antigens in tumors. However, this heterogeneity varies greatly among different breast cancer types and individuals, which may affect the efficacy of ADCs. The release and diffusion of payloads in the tumor microenvironment are complex, such as pH, enzyme activity, and redox status, which impair the exertion of the bystander effect. Additionally, ADCs may be prematurely degraded in the bloodstream, leading to the clearance of payloads and elevated toxicity [77,81]. Hence, it remains an overwhelming challenge to balance the benefits of the bystander effect and the off-target toxicity. Further research is needed to gain more ADCs with bystander effects and optimize the designs to enhance their efficacy and safety in the future.

**Table 3:** The ADC drugs for HER2-positive breast cancer.

ADC Drugs	Phase	Components	Bystander Effect	Refs.
Trastuzumab emtansine (T-DM1)	Approved	Trastuzumab + thioether linker +tubulin inhibitor DM1	×	[77,78,79,80]
Trastuzumab deruxtecan (T-DXd)	Approved	Trastuzumab + tetrapeptide linker +topoisomerase inhibitor DXd	√	[81,82,83,84]
RC48	Phase III (NCT04400695)	Hertuzumab + valine-citrulline linker +tubulin inhibitor MMAE	√	[86]
A166	Phase II (NCT05346328)	Trastuzumab + valine-citrulline linker +tubulin inhibitor Duo-5	√	[87]
SHR-A1811	Phase III (NCT06057610)	Trastuzumab + tetrapeptide linker +topoisomerase inhibitor SHR9265	√	[88]

## 6. BsAb Drugs

Prepared through technologies such as cell fusion, recombinant DNA, and protein engineering, bsAb is an artificial antibody that can simultaneously or successively specifically bind to two antigens or two different epitopes of the same antigen to enhance its binding specificity and reduce adverse reactions caused by off-target toxicity. This drug enables the attachment of effector cells, like immune cells or cytokines, to tumor cells and enhances the killing effects, thereby, theoretically, exerting a better therapeutic effect than mAb drugs [89]. BsAb significantly enhances the signal blocking ability and anti-tumor activity by simultaneously targeting different epitopes of HER2 or in combination with other receptors (such as HER3). Currently, some bsAb drugs have demonstrated clear clinical potential, while some bsAb drugs remain at the early validation stage (Table 4). Future research directions include optimizing combined treatment strategies (such as chemotherapy or immunotherapy) and expanding indications to the population with low HER2 expression.

### 6.1. BsAb

Anbenitamab, Zanidatamab, and TQB2930 target the ECD II and IV of HER2 to doubly block the HER2 signaling pathway, similar to the synergistic effect of combining trastuzumab with pertuzumab. The dual epitope binding can trigger stronger signal blocking and an internalization effect over the mAb resistance mechanism. The clinical trials preliminarily demonstrated that these three bsAb drugs were well tolerated and achieved comparable efficacy as trastuzumab and pertuzumab in pretreated patients with HER2-positive breast cancer [90,91]. Some ongoing clinical trials are exploring their combination regimens with chemotherapy to further verify their potential in patients who have failed in multiple lines of treatment.

### 6.2. BsADC

BsADC combines the advantages of bsAb and ADC by simultaneously targeting two antigens or different epitopes of the same antigen, which can significantly improve targeting, anti-tumor activity, and safety. JSKN003 is a novel ADC targeting HER2 dual epitopes. Alphamab Oncology developed this site-specific modified ADC with a DAR value of approximately 4 through enzymatic catalysis and click chemical reaction of the heavy chain sugar groups of anbenitamab [92]. JSKN003 can bind to HER2 on the surface of tumor cells and release topoisomerase inhibitors through endocytosis, thereby exerting anti-tumor effects. JSKN003 has better serum stability and a stronger bystander killing effect compared with similar ADC drugs, effectively expanding the therapeutic window. JSKN003 is currently undergoing multiple clinical trials at different stages, and the research results have demonstrated good tolerance and safety. It has shown significant efficacy in patients with various advanced solid tumors who have previously received multiple lines of systemic anti-tumor therapy, especially in HER2-expressing breast cancer, and has entered the Phase III clinical stage. ZW49, composed of zanidatamab + disulfide bond linker + tubulin inhibitor auristatin, is another bsADC that has entered the clinical stage (Phase I) for patients with HER2-positive cancers; however, the clinical data did not meet expectations, and most patients experienced treatment-related adverse events.

Judging from the current research and development situation, perhaps due to the fact that the mature target HER2 has been fully verified in various therapies, and considering the low research and development risk and high success rate, the main bsADC drugs under research and development at present are mainly HER2 dual-antibody ADCs. In addition to dual-epitope bsADC with the same antigen, there are also research and development paths targeting two different antigens on the same cancer cell. The study of Zong HF et al. constructed a HER2/HER3-targeting bsADC (DAR 2.89) and displayed high selectivity and better internalization, which suppressed breast cancer cells in vitro with a comparable efficacy of HER2- and HER3-monospecific ADC combined injection [93]. Alternatively, CD63, the prolactin receptor, and amyloid beta precursor-like protein 2 are selected as promising candidate receptors for discovering novel bsADC drugs dual-targeting HER2; they would have been taken into therapeutic consideration for HER2-positive breast cancer if they bore the capacity to cover single-target combined therapy [94,95,96].

**Table 4:** The bsAb drugs for HER2-positive breast cancer.

BsAb Drugs	Targets	Components	Phase	Ref.
Anbenitamab	HER2 ECD II/IV	BsAb	Phase III (NCT06747338)	[90]
Zanidatamab	HER2 ECD II/IV	BsAb	Phase III (NCT06435429)	[91]
TQB2930	HER2 ECD II/IV	BsAb	Phase II (NCT06202261)	-
JSKN003	HER2 ECD II/IV	Anbenitamab + gly-gly-phe-gly linker +topoisomerase inhibitor	Phase III (NCT06846437)	[92]
ZW49	HER2 ECD II/IV	Zanidatamab + disulfide bond linker +tubulin inhibitor auristatin	Phase I (NCT03821233)	-
HER2/HER3 BsADC	HER2 HER3	BsAb + maleimidocaproyl/valine-citrulline/ PABA linker + tubulin inhibitor MMAE	Preclinical study	[93]

## 7. Discussion

In recent years, with the development of molecular biology, targeted therapy for carcinoma has become a hotspot in clinical tumor treatment. Clinical practice has confirmed the feasibility of tumor-targeted therapy theory, and compared with traditional radiotherapy and chemotherapy, tumor-targeted therapy has strong specificity, significant therapeutic benefit, and relatively small toxic and side effects. However, it is very important to select an effective molecular target for tumor-targeted therapy, just as HER2 in response to breast cancer. HER2 plays a significant role at all stages of normal cellular development, but its overexpression and mutation can trigger the occurrence and metastasis of carcinoma. The status of gene amplification and overexpression makes HER2 a promising tumor therapeutic target in various types of human cancers. Studies have shown that the HER2-overexpressed phenotype is highly invasive and represents approximately 15–20% of all breast cancers, accompanied by a short disease-free survival period and a poor prognosis [8]. Since HER2 abnormality is very common in breast cancer, the current National Comprehensive Cancer Network (NCCN) guidelines and Chinese Society of Clinical Oncology (CSCO) guidelines clearly recommend that HER2 testing should be conducted for breast cancer therapy, comprehensively determined by combining HER2 protein level (immunohistochemistry) and amplification level (fluorescence in situ hybridization/sequencing). Therefore, to determine whether breast cancer patients can use HER2-targeted drugs is of vital importance, which requires the precise testing and evaluation of HER2 protein expression and gene amplification status in breast cancer. In the past, the positive standard for HER2 was set by trastuzumab. With the advancement towards newer clinical efficacy standards, the detection standards for biomarkers will face continuous iterations and optimizations, providing further clinical evidence for the precise treatment of future patient populations.

Looking back at the development history of HER2-targeted drugs, since trastuzumab was launched as the first HER2-targeted mAb in 1998, it has not only changed the treatment landscape for HER2-positive breast cancer patients but also promoted clinical transformation. However, since some patients taking this drug may develop drug resistance or relapse, the small-molecule TKI drug lapatinib was introduced in 2007, combining with capecitabine for the treatment of HER2-positive metastatic breast cancer that failed trastuzumab. In 2013, the first ADC for treating solid tumors, T-DM1, was launched on the market and acted like an upgraded version of trastuzumab, not only achieving good therapeutic effects but also having fewer toxic reactions, making it more tolerable for patients. Since then, HER2-targeted drugs have occupied a place in the fields of mAbs (trastuzumab, pertuzumab, margetuximab, biosimilars…), TKIs (lapatinib, neratinib, tucatinib, pyrotinib, afatinib, dacomitinib…), ADCs (T-DM1, T-DXd, RC48, A166, SHR-A1811…), and later bsAbs (anbenitamab, zanidatamab, TQB2930…) and bsADCs (JSKN003, ZW49, HER2/HER3 BsADC…), which have flourished in multiple areas and entered a new situation in various tumors such as breast cancer, gastric cancer, and non-small cell lung cancer. Targeted drugs for HER2-positive breast cancer are developing rapidly, which improve the prognosis and life quality of patients diagnosed with early and metastatic stages. We have, in detail, reviewed the drug action mode, indication, administration, efficacy, strengths, and challenges of HER2-targeted drugs in this article; however, there are still many unresolved issues for HER2-targeted drugs, and corresponding clinical trials are still ongoing to further optimize the efficacy for the patient population.

The timely detection of drug resistance is very crucial for treatment management, followed by several commonly used monitoring methods. Imaging examinations: regular computerized tomography, magnetic resonance imaging, biomarker testing: HER2 levels, HER2 signaling pathway activity (Ras/Raf/MEK/MAPK, PI3K/AKT, PLC/PKC, and STAT5), and sequencing: gene mutations and changes can provide doctors with important information and specific mechanisms about drug resistance. MAbs have become a cornerstone in the treatment of HER2-positive breast cancer since their launch, suitable for neoadjuvant, adjuvant, and advanced treatment. The rise of mAb biosimilars and the innovation of bsAbs have also driven market competition and price decline. The oral TKIs can penetrate the blood–brain barrier and have unique advantages for patients with brain metastasis. Meanwhile, with the continuous iteration and update of technology, the progress of ADC drugs has been making continuous breakthroughs. The excellent clinical performance has attracted widespread attention to this field, and ADC drugs have entered a period of rapid development. Therefore, the targeted drugs for HER2-positive breast cancer have evolved from single targeting (mAbs) to combination therapy and multi-mechanism innovation (chemotherapy, TKIs, ADC, bsAbs) for various indications. In the future, more emphasis will be placed on precision, individualization, and accessibility. Given that each patient’s condition and response to treatment are unique, individualized treatment plans are crucial for managing drug resistance. Based on the specific conditions of patients, doctors should tailor an optimal treatment plan by integrating various combinations such as surgery, radiotherapy and chemotherapy, targeted therapy, immunotherapy, and new drugs in clinical trials, in order to enhance therapeutic effect and overcome drug resistance.

As rising star drugs, the design of ADC drugs aims to deliver cytotoxins to HER2-positive breast cancer cells through antibody targeting, thereby reducing toxicity to normal tissues. The development of ADC drugs has undergone three generations of technological upgrades, with the continuous optimization of therapeutic effects. Particularly, ADC drugs targeting HER2-positive breast cancer cells have made breakthroughs in aspects such as targets, antibodies, linkers, payloads, and coupling methods. Clinically, there are higher requirements for the next-generation ADC drugs, which need to have a larger therapeutic window and improve drug resistance. Due to the complex structure of ADCs and the multiple steps involved in their action mode, drug resistance may occur at any level, starting from antigen expression and recognition, ADC internalization and degradation, to the release of cytotoxic drugs and cell death regulation. Considering mAbs, cytotoxic drugs, and linkers, the mechanisms of T-DM1 resistance are more complex than trastuzumab itself. The abnormal antigen (low expression, high heterogeneity, mutation, modification, truncation, dimerization), failed payloads (DAR), failed internalization and transport, impaired lysosomal function, and excessive drug excretion can trigger T-DM1 resistance [97]. Moreover, topoisomerase 1 mutations occur after T-DXd treatment, but more clinical datasets are required due to the small number of cases and unclear impact of mutated topoisomerase 1 on T-DXd efficacy [98]. Therefore, comprehensive improvement and optimization need to be carried out in the following multiple aspects. Firstly, regarding antibody structure, most of the antibodies approved for ADC mainly come from IgG1, IgG2, and IgG4 due to high affinity and long circulation half-life, while IgG1 and its derivatives are widely applied in most anti-HER2 ADCs (T-DM1 and T-DXd) with strong ADCC effects [99]. Further, bsAb is designed to recognize and bind to two different epitopes or antigens, which provides new and promising strategies for the development of anti-HER2 ADCs (such as JSKN003 and ZW49) [92]. Secondly, regarding novel payload, the payloads are not limited to tubulin inhibitors and topoisomerase inhibitors, but also include protein toxins, radionuclides, ribosome inhibitors, siRNA, and immunostimulants [100,101]. We need to enhance the hydrophilic ability of payloads during modification to avoid ADC aggregation. In addition, dual-payload ADCs are also under consideration, but an appropriate DAR value is needed. Thirdly, novel linker: From the perspectives of pharmacokinetics and pharmacodynamics, the new linkers must be stable enough to circulate in blood and not be lysed prematurely, avoiding off-target toxicity. When internalized, the linkers should allow effective release of payloads. Importantly, the hydrophobic property is a key factor to suppress ADC aggregation in response to the linker [102]. Further, regarding bsADCs and non-internalized ADCs, compared with traditional ADCs, the unique dual epitope/target binding mode of bsADCs not only allows binding to co-expressed antigens in solid tumors to enhance selectivity, but also significantly improves internalization. Mabs usually have difficulty diffusing into solid tumors due to the antigen barrier; therefore, non-internalized antibodies can be developed for ADCs, which directly release payload outside tumor cells in the microenvironment and then diffuse inside to kill tumor cells [103]. These unique advantages make bsADCs and non-internalized ADCs an important force in the next-generation ADC field. However, the immunogenicity and off-target risks remain overwhelming challenges for bsADCs and non-internalized ADCs, respectively, and await further exploration in more preclinical models and clinical trials.

The resistance mechanism of HER2-positive breast cancer targeted drugs is very complex and involves numerous signaling pathways. Apart from the specific trends highlighted above, alternative pathways should not be ignored. Studies have shown that HER3 is highly expressed in the primary and metastatic tumors of breast cancer, or is up-regulated after HER2 inhibition [104]. Therefore, deeply understanding the compensatory activation process of HER3 and developing HER2/HER3 co-targeted drugs are of great clinical significance and will provide a new avenue for patients with HER2-positive advanced breast cancer who have failed multiple lines of treatment. Notably, intelligent nano-delivery carriers have attracted considerable interest in achieving precise drug release due to their potential to respond to the tumor microenvironment via pH, photothermography, or specific enzymes [105,106]. In the future, addressing the obstacles posed by nano-vectors may thus present a challenging and inevitable undertaking to enhance the safety and efficacy of anti-HER2 drugs.

## 8. Conclusions

Collectively, after decades of development, HER2-targeted drugs have made many encouraging advancements in the discovery process of mAbs, TKIs, ADCs, and bsAbs. The drug action mode, indication, administration, efficacy, strengths, and challenges of these HER2-targeted drugs have been summarized in this article. The continuous innovation of HER2-targeted drugs brings more thorough and precise efficacy, reduces the toxic effects, drug resistance, and recurrence risk, and also provides more diverse options for HER2-positive breast cancer patients. With the gradual increase in the number of new cases of breast cancer worldwide, HER2 has long been a popular target for the treatment of breast cancer. The demand from patients for the next generation of anti-HER2 drugs is bound to trigger a new round of research and development boom. Although the survival rate of HER2-positive breast cancer has been greatly improved, more precise drug efficacy, lower side effects, and drug resistance remain urgent problems to be solved in further clinical practice.

## Data Availability

No new data were created or analyzed in this study.

## Abbreviations

ADCC	Antibody-dependent cell-mediated cytotoxicity
ADCs	Antibody-drug conjugates
BsAbs	Bispecific antibody
CDK	Cyclin-dependent kinase
CSCO	Chinese Society of Clinical Oncology
DAR	Drug-antibody ratio
Duo-5	Duostatin-5
EGFR	Epidermal growth factor receptor
ER	Estrogen receptor
ECD	Extracellular domain
FDA	Food and Drug Administration
HB-EGF	Heparin-binding epidermal growth factor-like growth factor
HER	Human epidermal growth factor receptor
IDFS	Invasive disease-free survival
IGF-1R	Insulin-like growth factor 1 receptor
MAbs	Monoclonal antibodies
MMAE	Monomethyl auristatin E
NCCN	National Comprehensive Cancer Network
NRG	Neuregulin
OS	Overall survival
PR	Progesterone receptor
PFS	Progression-free survival
RTK	Tyrosine kinase receptor
TKIs	Tyrosine kinase inhibitors
TNBC	Triple negative breast cancer
TGF-α	Transforming growth factor-α
T-DXd	Trastuzumab deruxtecan
T-DM1	Trastuzumab emtansine
